# The Treatment of Very Large Traumatic Bone Defects of the Tibia With a Polycaprolactone-Tricalcium Phosphate 3D-Printed Cage: A Review of Three Cases

**DOI:** 10.7759/cureus.66256

**Published:** 2024-08-06

**Authors:** Anna Lodewijks, Taco Blokhuis, Martijn van Griensven, Martijn Poeze

**Affiliations:** 1 Department of Traumatology, Maastricht UMC+ (University Medical Center), Maastricht, NLD; 2 Institute of Nutrition and Translational Research in Metabolism (NUTRIM), Maastricht University, Maastricht, NLD; 3 Institute for Technology-Inspired Regenerative Medicine (MERLN), Maastricht University, Maastricht, NLD

**Keywords:** cage, nonunion, bone regeneration, scaffold, large bone defect, polycaprolactone-tricalcium phosphate, induced membrane technique

## Abstract

The need for an artificial scaffold in very large bone defects is clear, not only to limit the risk of graft harvesting but also to improve clinical success. The use of custom osteoconductive scaffolds made from biodegradable polyester and ceramics can be a valuable patient-friendly option, especially in case of a concomitant infection. Multiple types of scaffolds for the Masquelet procedure (MP) are available. However, these frequently demonstrate central graft involution when defects exceed a certain size and the complication rates remain high. This paper describes three infected tibial defect nonunions with a segmental defect over 10 centimeters long treated with a three-dimensional (3D)-printed polycaprolactone-tricalcium phosphate (PCL-TCP) cage in combination with biological adjuncts.

Three male patients, between the ages of 37 and 47, were treated for an infected tibial defect nonunion after sustaining Gustilo grade 3 open fractures. All had a segmental midshaft bone defect of more than 10 centimeters (range 11-15cm). First-stage MPs consisted of extensive debridement, external fixation, and placement of anterior lateral thigh flaps. Positive cultures were obtained from all patients during this first stage, which were treated with specific systemic antibiotics for 12 weeks. The second-stage MP was carried out at least two months after the first stage. CT scans were obtained after the first stage to manufacture defect-specific cages. In the final procedure, a custom 3D-printed PCL-TCP cage (Osteopore, Singapore) was placed in the defect in combination with biological adjuncts (BMAC, RIA-derived autograft, iFactor, and BioActive Glass). Bridging of the defect, assessed at six months by CT, was achieved in all cases. SPECT scans six months post-operatively demonstrated active bone regeneration, also involving the central part of the scaffold. All three patients regained function and reported less pain with full weight bearing.

This case report shows that 3D-printed PCL-TCP cages in combination with biological adjuncts are a novel addition to the surgical treatment of very large bone defects in (infected) post-traumatic nonunion of the tibia. This combination could overcome some of the current drawbacks in this challenging indication.

## Introduction

The treatment of large bone defects after trauma, infection, or malignancy can be challenging [1]. Regeneration of bone in large and very large bone defects is limited by the natural capacity of bone, and therefore, a high failure rate is shown in the literature [2]. Failure to treat these defects effectively, for example in case of a concomitant infection, may result in amputation of the affected extremity [3]. Several techniques have been suggested for the treatment of large bone defects, one of which is the induced membrane or Masquelet procedure (IMT or MP) [4]. This procedure consists of two surgical steps. During the first step, the nonunion is debrided, stabilized, and filled with a cement spacer. The cement induces the formation of a membrane around the spacer. After removal of the cement spacer in the second surgical step, the induced membrane, or ‘neoperiosteum’ that surrounds the remaining cavity, is filled with grafting materials, providing a biomechanical and biological environment for bone regeneration. Reported success rates are high, between 82 and 100% [5], although in these studies defects were filled with autograft as a stand-alone. In very large defects, where autografting does not provide quantitative and/or qualitative sufficient material, other osteoconductive or osteoinductive materials need to be added [6]. Although a wide variety of different materials is commercially available, the ideal solution is still the subject of ongoing discussion, especially in large segmental defects. For osteoconductive materials, both the composition and physical application are being studied to optimize their use. For application of osteoconductive materials in very large defects, three-dimensional (3D) printing is a promising technique to produce custom-made blocks with properties that improve bone healing.

3D printing of porous cage graft extenders has already been described for bridging very large defects, with the aim of increasing stability, bone growth, and union rates [7]. Titanium alloy is one of the materials previously used for these cages. This has been shown to have good osteoconductive properties [8]. In contrast, titanium does not have any osteoinductive properties and is non-absorbable. Additionally, instead of incorporation of the material into the bone, the bone merely grows around it, which may delay the consolidation process and may influence the biomechanical properties of the regenerated bone over time [9,10]. The challenge is to create a graft that is biodegradable, meaning it will be fully incorporated in the newly formed bone over time, but initially provides enough structural support in large bone defects. In addition, the material has to provide an optimal environment to promote bone regeneration with cell growth and proliferation. Polycaprolactone is a biodegradable polyester that was discovered in the 1930s and has since been used for a variety of medical purposes, such as sutures and scaffolds. The material provides enough stability to be used as a bone scaffold that slowly degrades during two to four years [11]. Tricalcium phosphate (TCP) is a widely used ceramic with well-known osteoconductive properties. Since TCP degrades faster than PCL, the combination of the two materials may be advantageous in bone regeneration [12-14]. The combination of PCL and TCP has already been shown to promote osteogenic differentiation in in vitro studies in mesenchymal stem cells.

This paper will discuss three clinical cases with a segmental bone defect in which a PCL-TCP 3D printed cage (Osteopore, Singapore) was applied in combination with bone marrow aspirate concentrate (BMAC). This article was previously presented as a poster at the ICORS World Congress of Orthopaedic Research in September 2022 and the E.S.T.R.O.T. Congress in September 2022.

## Case presentation

Methods and manufacturing

**Figure 1:**
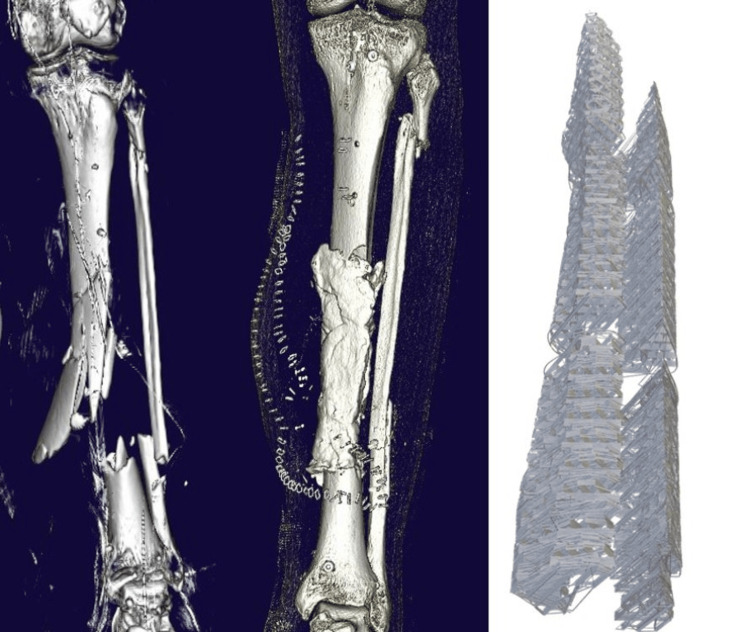
Three-dimensional reconstructions of the CT images of the initial injury and after the first stage IMT, and the virtually designed cage. IMT: Induced membrane procedure

After the first-stage procedure, the defect is filled with a cement spacer. A CT scan of the defect is made shortly after the procedure to evaluate the exact defect dimensions and stabilization method. This CT scan, together with imaging of the healthy contralateral side, is used as a blueprint for the 3D-printed scaffold (Figure 1). The Digital Imaging and Communications in Medicine (DICOM) file is used to create a 3D model that can be exported as a Standard Tessellation Language (STL) file, which is then further processed in modeling software for mesh printing. The 3D-printed scaffold is produced by Osteopore® International Pte. Ltd. (Singapore). The manufacturing process will take six to eight weeks, which is similar to the time between the first and second stages of the procedure.

The scaffold is printed in a honeycomb structure using a fused deposition modeling printing method. It is made into multiple components, enabling it to be fitted inside the bone defect around the osteosynthetic material. The finished scaffold consists of 80% TCP and 20% PCL, with a porosity of 80% to allow loading with autografting material and bone growth inside the scaffold. The printed scaffold is sterilized using irradiation.

Case 1

A 47-year-old man was brought into the emergency department after a high-energy motorcycle accident. The patient sustained a Gustilo grade 3b open tibia fracture in combination with a two-part fibula fracture and an ipsilateral Lisfranc injury. He received two grams of cefazolin and 560 milligrams of gentamycin according to hospital protocol. The patient underwent extensive wound debridement and an external fixator was placed spanning the affected lower leg and foot. Post-operative care included three days of intravenous (IV) cefazolin. Thirteen days after the injury, a definitive fixation of the tibia using a reamed intramedullary nail was performed, as well as debridement of part of the anterior compartment and non-vital bone tissue, creating a segmental defect in the tibia of 15 centimeters. An antibiotic-loaded cement spacer (Refobacin, Zimmer Biomet) was placed to fill this defect temporarily. The Lisfranc injury was treated by open reduction and internal fixation. The fibular fracture was treated by plate fixation. Soft tissue coverage was performed by the plastic surgeon using an anterolateral thigh flap (ALT). Post-operative care consisted of intravenous antibiotics and non-weight-bearing physical therapy. Post-operatively, the proximal rim of the ALT flap did not heal, forming a fistula. Because of this fistula, three months after the placement of the tibial nail and cement spacer, the nail and cement were exchanged and the fistula was debrided instead of the planned second stage of the Masquelet procedure. Cultures showed Staphylococcus aureus and S. epidermidis, for which the patient received vancomycin and flucloxacillin intravenously for two weeks. Due to the additional debridement operation, exactly seven months after the initial injury a definitive fixation of the fracture was performed. This included the placing of an antibiotic-coated tibial intramedullary nail (Protect, Johnson and Johnson), placement of a 3D printed PCL/TCP cage (Osteopore, Singapore), and filling of the defect using cancellous bone harvested from the left femur using a reamer intramedullary aspirator (RIA, Johnson and Johnson), as well as bone marrow aspirate concentrate (BMAC), which was obtained from the iliac crest and processed according to the manufacturer’s instructions (Arthrex, Florida, USA). The skin was closed by transposition of the ALT and placement of a full-thickness graft (FTG). For a total of two months oral antibiotics, cotrimoxazole, and rifampicin were continued. There were no post-operative complications after this procedure. Between hospital admissions, the patient followed a revalidation program in a rehabilitation clinic. No additional procedures for consolidation were necessary.

**Figure 2:**
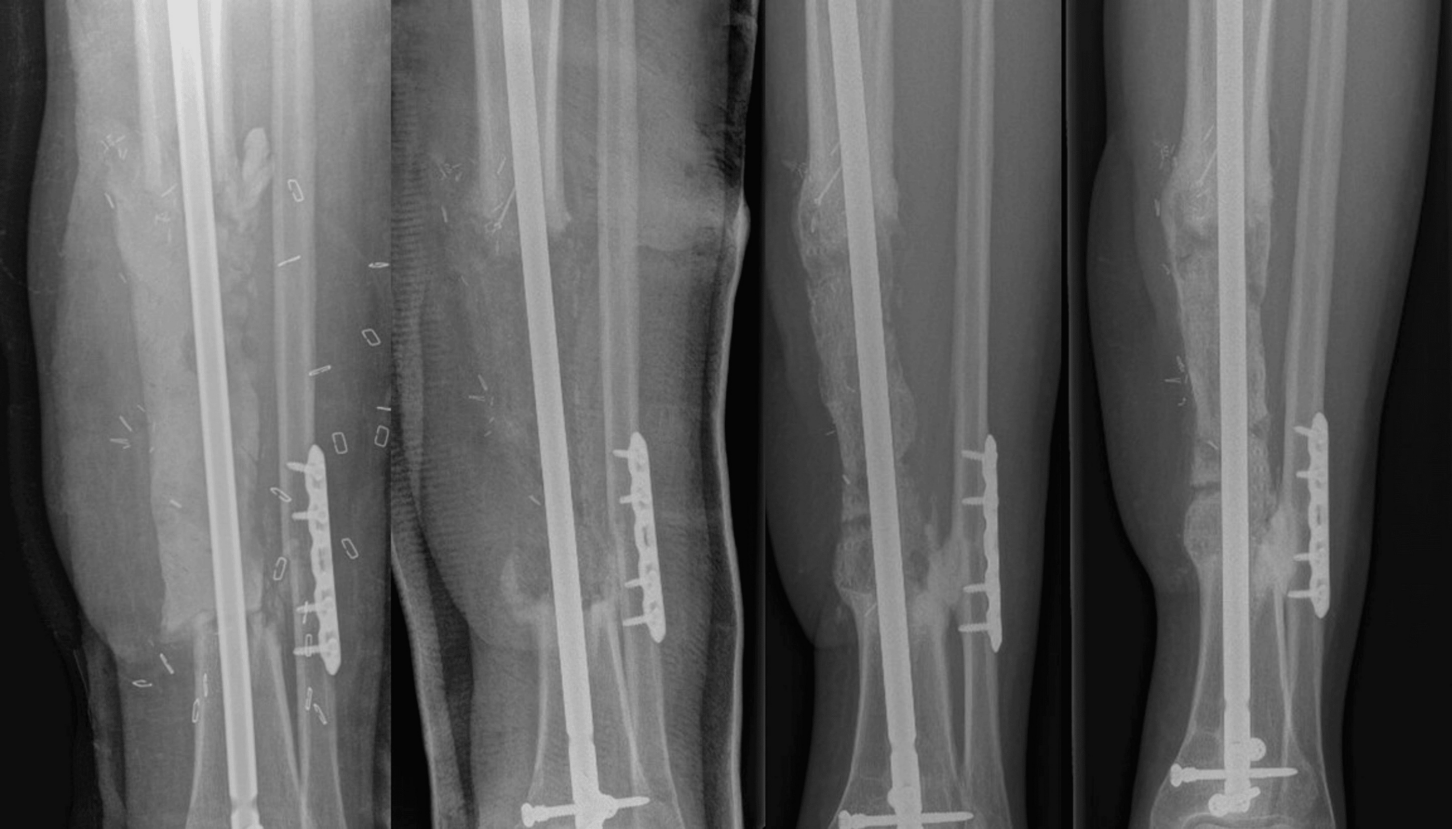
Case 1: One day, three months, one year, and two years after the second-stage IMT. IMT: Induced membrane procedure

**Figure 3:**
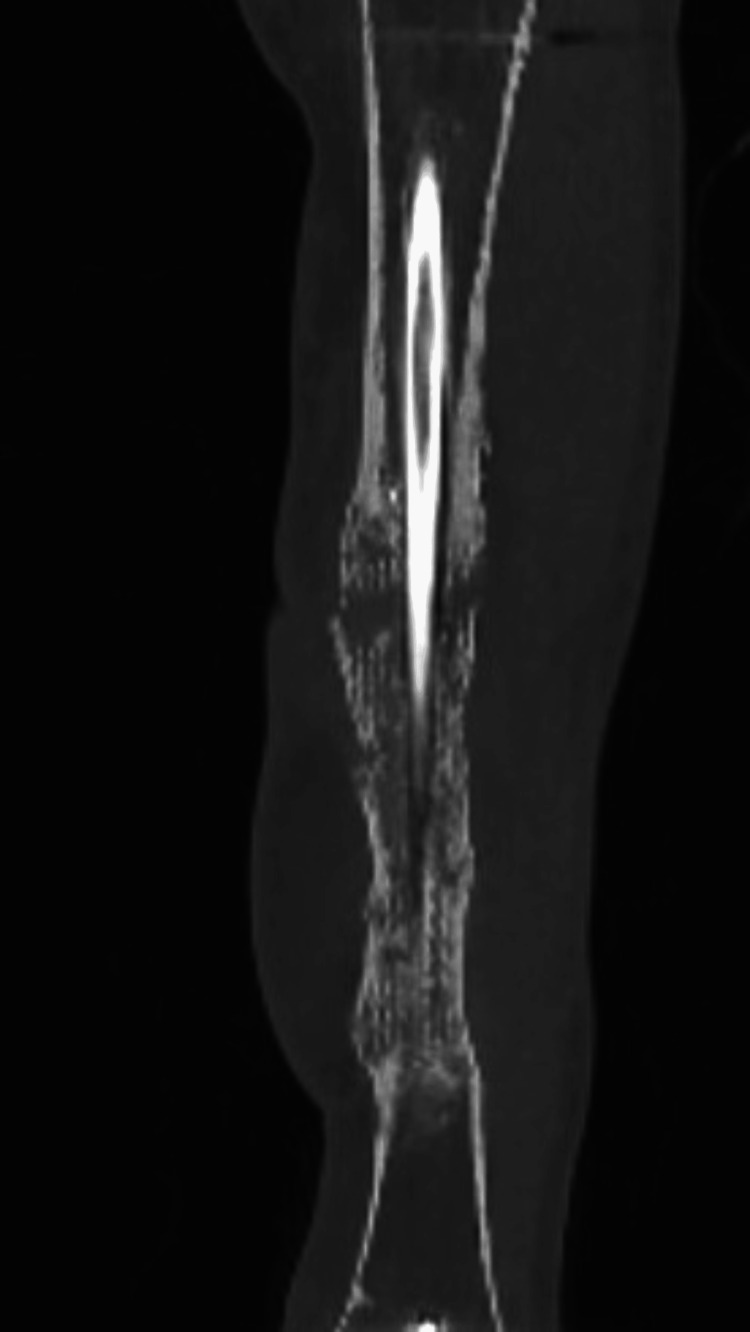
Case 1: Low-dose CT one and a half years after the second stage IMT. IMT: Induced membrane procedure

One year after the final surgery, the patient had a difference in leg length of three centimeters, for which the patient wore adjusted shoes. There is some manageable pain in the midfoot, and no pain surrounding the initial site of the crus fracture. Plain radiographs and low-dose CT show ongoing bone regeneration at the defect site over the 3D-printed cage and incorporation of the cage (Figure 2, 3).

Case 2

**Figure 4:**
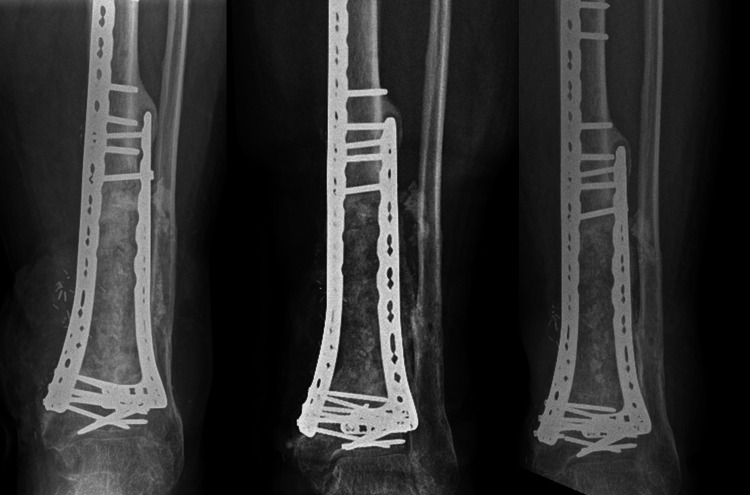
Case 2: One day, three months, and one year after the last-stage IMT. IMT: Induced membrane procedure

**Figure 5:**
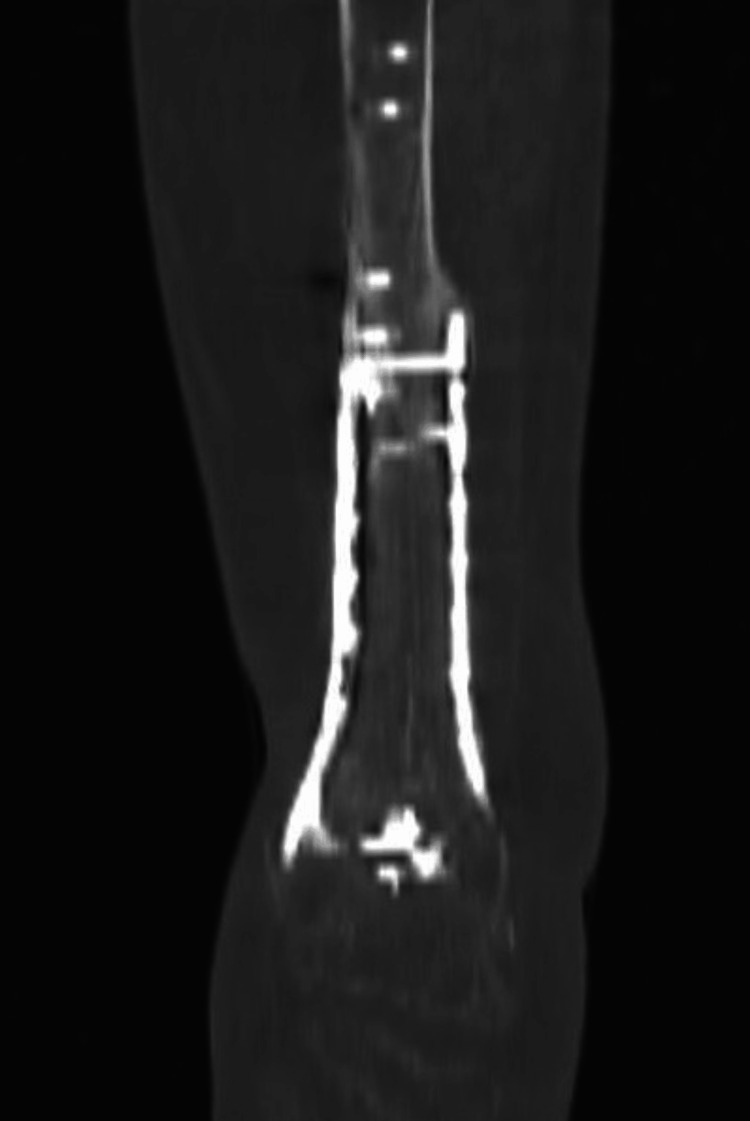
Case 2: Low-dose CT one year after the second-stage IMT IMT: Induced membrane procedure

The second patient was a 46-year-old man who was referred to our institution after suffering a Gustilo grade 3A open distal tibial fracture of the left leg after a fall of a forklift, primarily treated at another hospital. At first presentation in our institution, the trauma was eight months ago, and he had previously been treated with an external fixator followed by a plate fixation, ALT flap, and split skin graft one week after the injury. Radiographs did not show any signs of consolidation and a PET-CT showed a fistula from the defect to the skin and increased activity surrounding the implant. After surgical removal of the implant and debridement of the nonunion, the remaining segmental defect of 8 centimeters was filled with cement and the fracture was stabilized using an external fixator. Sonification and tissue cultures showed a Staphylococcus aureus and Pseudomonas aeruginosa, for which the patient received piperacillin/tazobactam intravenously. A second surgical procedure followed two months later, using plate fixation and filling of the defect using Bioglass granules (Bonalive, Finland) and BMAC [15]. Radiographs did now show adequate consolidation of the proximal docking site for which a percutaneous application of BMAC took place eight months later. Three months after surgery nonunion of the proximal part of the defect persisted. Another debridement took place leaving a bone defect of 10 cm, which was filled with cement. Cultures did not identify bacteria. Seven months later the final second stage Masquelet took place, using a 3D printed PCL/TCP cage. This surgery was performed three years and five months after the primary injury. The 3D-printed cage was augmented with cancellous bone that was harvested with the RIA technique. In addition, BMAC and a bioactive peptide (iFactor, Cerapedics, USA) were added [16]. Radiographs three months and one year after the surgery show incorporation of the graft material and ongoing remodeling of the defect (Figure 4). A low-dose CT scan confirmed the incorporation at a one-year follow-up (Figure 5). Apart from some complaints of pain during walking, the patient is making progress and the pain is bearable. No additional procedures for consolidation were necessary.

Case 3

**Figure 6:**
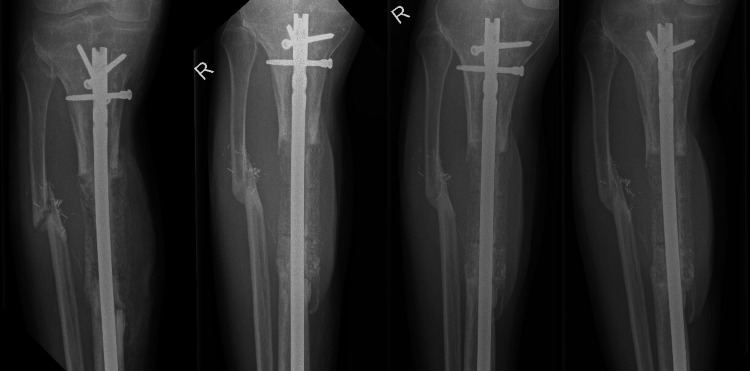
Case 3: One day, five months, one year, and one and a half years after the second-stage IMT. IMT: Induced membrane procedure

A male patient, 37 years old, was referred to our institution after a motorcycle accident eight months prior. At that time, the patient suffered a Gustilo grade 1 open lower tibia fracture that was initially treated with a tibial nail (T2, Stryker) and cerclage wiring. This was complicated by a wound infection, causing a bacteremia for which IV antibiotic treatment was given. At first presentation, there was an apparent wound defect showing an avascular part of the tibia surrounded by pus. Radiographs showed signs of infection and no signs of consolidation with avital bone distal from the cerclage. Subsequently, a first-phase MP was performed, leaving a defect of 10 cm in the tibia that was filled with a cement spacer, and the tibia was stabilized with an external fixator. The wound was temporarily closed using a synthetic skin replacement. One month later a second first-stage MP was performed and the soft tissue was covered with an ALT flap. Cultures showed Enterobacter cloacae and Haemophiles parainfluenza, for which meropenem was prescribed. Following the bacterial cultures, a switch to vancomycin was made. A second phase of MP took place three months later. After the introduction of an intramedullary nail, a 3D-printed PCL/TCP cage that was augmented with BMAC, cancellous bone, and i-Factor, was placed. Follow-up showed progression of consolidation of the defect (Figure 6). One and a half years after the last surgery, the patient mobilizes with crutches due to pain around the knee and hip joint. No additional procedures were necessary to procure healing.

## Discussion

This case series describes three successful applications of PCL-TCP biodegradable bone scaffold cages in patients with a post-traumatic segmental bone defect of the lower extremity using the induced-membrane approach. Although the cages are combined in all cases with additives such as cells and autograft, the results are encouraging.

In all patients, incorporation of the grafting material was confirmed by a low-dose CT scan. The CT indicated full bony bridging of the defect and ongoing remodeling of the graft material.

The three cases describe the course of the treatment of large diaphyseal segmental bone defects. In addition to the substantial bone defects, infection was also present in all three cases. The successful outcome indicates that the chosen approach with a 3D-printed PCL/TCP cage supports the bone regeneration potential that is needed in these challenging cases. Fractures with two or more centimeters of bone loss are less likely to heal spontaneously and often need more extensive surgical treatment [2]. Although larger defects have been reported to heal spontaneously, the tibia is known to do the opposite [17,18]. Tibial fractures with a segmental defect size of more than two and a half centimeters are reported to develop a nonunion in over 50% of cases [19]. One of the anatomical risk factors is the long subcutaneous border of the front of the tibia. Because of this, blood supply to the tibia is limited compared to other long bones, thus increasing the chance of suffering a detrimental outcome in open fractures [20,21]. Open fractures of the tibia have a high risk of delayed- or nonunion, especially when coinciding with infectious complications. Apart from the trauma-induced size of a bone defect and soft tissue damage, the occurrence of infection with open fractures will increase the need for debridement, leaving even larger bone defects [22].

For a long time, amputation was considered a feasible treatment option for complex open, infected fractures. Because of the relatively good functional outcomes reported with early amputation and a shorter hospitalization time [23,24]. However, with improved understanding of bone biology and the introduction of additional treatment options for these large defects, amputation may be prevented more frequently. Additionally, a more recent study by Vincken et al. found better patient-reported functional outcomes in lower limb reconstruction over time than currently reported in amputees [25,26]. Furthermore, lifetime costs for amputation are significantly higher than reconstruction because of the prosthesis, long revalidation times, and secondary surgeries [27].

In this case series, limb reconstruction is performed using the induced membrane or the Masquelet technique is a successful procedure in treating fractures with bone loss due to the beneficial properties of the induced membrane. This membrane not only contains the grafting material and prevents resorption of the bone graft, but also provides an environment for enhanced vascularisation, which leads to an increase of osteogenic cells and growth factors at the fracture site [7]. A substantial number of studies have demonstrated the success of this procedure, although reported success rates are lower in larger defects [2]. However, the limited success rate in larger defects is seen in defects treated with autograft as stand-alone. Autograft is long known to be limited in volume, but also biological activity. When treating defect sizes over 10 cm in length the effectiveness of autograft should therefore be enhanced by the addition of other materials, such as synthetic grafting materials or bioactive peptides [28]. 

3D printing of porous cage graft extenders was performed to bridge very large defects, with the aim to increase stability, bone growth, and union rates and thereby decrease the number of reoperations [7]. The PCL-TCP cages used are biodegradable and incorporated themselves into the newly formed bone over time in the presented cases, a clear advantage over the cages made out of titanium previously [9]. The cages are used to augment the scaffolding of the autografts from the RIA with additional stimulation of bone regeneration with a combination of BMAC and other growth-inducing materials. The advantageous effects of adding cells and bioactive peptides to PCL-TCP have already been shown by others, but to our knowledge, this is the first report of this effect in humans [29,30].

## Conclusions

Multiple treatment strategies are known to be effective in tibial nonunion surgery, but success rates decrease when defects are large and especially when an infection is present. This paper describes three successful treatments of very large infected bone defects, ranging from 10 to 15 centimeters, combining the induced membrane technique with 3D-printed PCL-TCP cages. It shows that this treatment plan is a promising addition to the care of highly complex cases of infected nonunion of the tibia. Follow-up radiographs of the defects all showed incorporation of the graft into the tibia. More important is that all patients were, and are still, making progress regarding mobilization and pain. More research, including more patients with a longer follow-up period, should be conducted to further explore the capacities of 3D-printed cages in this challenging condition, but the first results are encouraging. 
